# The Decline in the Birth-Rate

**Published:** 1913-09-13

**Authors:** Binnie Dunlop

**Affiliations:** Alexandra Court, S.W.


*To the Editor of* The Hospital.

### The Decline in the Birth-rate.

*To the Editor of* The Hospital.

Sir,—The medical profession, as you point out in your very useful article on "The Decline in the Birth-rate," has not been realising the lessons of vital statistics. As I have already suggested in your columns, the falling birth-rate in this and other countries has meant fewer deaths—not fewer inhabitants. So the important question is, as you say, "not the number of births, but the value of the births recorded." The artificial family limitation which has been going on since 1877 has been mainly practised by the richer classes. It seems to me most urgent —on humanitarian, eugenic, and social-unrest grounds, and in view of the ominously rising cost of living—that we immediately begin to encourage the married poor to avoid bringing more children into the world than they can themselves afford to feed, clothe, and house properly.—I am, Sir, etc.,

Binnie Dunlop, M.B., Ch.B.
Alexandra Court, S.W.

*Note.*—Owing to the pressure on our space we have had to hold over a letter on "Dissenters and Medical Authority."

